# Optimization of Reduced Glutathione Production by a *Lactobacillus plantarum* Isolate Using Plackett–Burman and Box–Behnken Designs

**DOI:** 10.3389/fmicb.2017.00772

**Published:** 2017-05-09

**Authors:** Lamiaa A. Al-Madboly, Eman G. Khedr, Safaa M. Ali

**Affiliations:** ^1^Department of Pharmaceutical Microbiology, Faculty of Pharmacy, Tanta UniversityTanta, Egypt; ^2^Department of Biochemistry, Faculty of Pharmacy, Tanta UniversityTanta, Egypt; ^3^Department of Nucleic Acid Research, Genetic Engineering and Biotechnology Research Institute, City for Scientific Research and Technology ApplicationsAlexandria, Egypt

**Keywords:** glutathione, *L. plantarum*, Placket–Burman, Box–Behnken, optimization

## Abstract

In this work, we aim to optimize the production of reduced glutathione (GSH) synthesized intracellularly by a food-grade microorganism through a statistical approach. Using a colorimetric method, 25 *Lactobacillus plantarum* isolates were screened in an attempt to find a GSH-producing strain. It was found that 36% of the tested isolates showed positive result. Isolate (L_7_) was found to produce 152.61 μM glutathione per gram which was the highest amount produced intracellularly. Accordingly, the later isolate was selected for the optimization process using Plackett–Burman and Box–Behnken designs. Temperature, amino acids, and urea were found to be the most significant independent variables. Following data analysis, the composition of the optimized medium was De Man-Sharp-Rogosa broth as a basal medium supplemented with NaCl (5%), H_2_O_2_ (0.05%), sodium dodecyl sulfate (0.05%), amino acids (0.0281%), and urea (0.192%). The pH of the medium was adjusted to 8 and incubated for 24 h at 40°C. The GSH amount was increased by 10-fold (851%) using the optimized medium. Hence, our optimization design estimated the biotechnological potential of *L. plantarum* (L_7_) for the production of GSH in the industry.

## Introduction

Glutathione is a tripeptide thiol composed of γ-glutamylcysteinylglycine (GSH) which is found in all eukaryotes and some prokaryotes such as probiotics. GSH is involved in many physiological processes and the general functions can be summarized in three ways, i.e., GSH serves as an antioxidant, an immunity booster, and a detoxifier in eukaryotes. First, the strong electron-donating capability of GSH and the relatively high intracellular concentration enable the maintenance of a reducing cellular environment. This makes GSH an important antioxidant for protecting DNA, proteins and other biomolecules against oxidative damage produced by reactive oxygen species. Second, GSH has potent antiviral activity in addition to the enhancement of immunity. Finally, GSH is significant in detoxification reactions via glutathione-*S*-transferase. Thus, GSH is a powerful defense molecule generated in prokaryotes and eukaryotes (Halliwell and Gutteridge, 1992; Pastore et al., 2003).

Different strategies have been developed to maintain high cellular levels of GSH. The most common strategy is the administration of GSH or its precursors. One innovative approach is the consumption of living microorganisms known as probiotics, i.e., live dietary supplements possibly administering specific health benefits to the host. Some probiotics have antioxidative systems to maintain free radicals below toxic levels (Farr and Kogoma, 1991). Glutathione production was detected among yeast (*Saccharomyces cerevisiae* and *Candida utilis*) as well as lactic acid bacteria, including Bifidobacterium, *Lactobacillus casei* HY2782, *L. acidophilus* ATCC 4356, *L*. *plantarum*, *L. fermentum*, *Lactococcus* *lactis* spp. *cremoris*, *Streptococcus thermophiles*, *Leuconostoc* *mesenteroides* spp. *cremoris*, *Lc.* *lactis* spp. *lactis*, micrococci, and pediococci in concentrations ranging from 6 nmol/g to 51 μmol/g. How different media types and cellular growth phases affect the intracellular levels of GSH have been also reported in the literature (Murata and Kimura, 1982; Fernandes and Steele, 1993; Li et al., 2004; Yoon and Byun, 2004).

Glutathione can be produced either enzymatically or by fermentation. Currently, the most important method used for industrial production is the fermentation using yeast such as *S. cerevisiae* or *C. utilis*. Some additional work addressing GSH production in bacteria has been conducted. For example, glutamylcysteine synthetase variants that were desensitized for GSH feedback inhibition had been isolated and cloned, resulting in higher GSH levels. Additionally, *Lc. lactis* has been used for GSH production even though it has no endogenous GSH (Chi-Hsien et al., 1999; Wei et al., 2003; Li et al., 2004; Masip et al., 2006). Therefore, the development of an industrial fermentation process requires medium optimization and our work is considered to be the first report on the optimization of GSH production using *L. plantarum* isolate. Optimization using a single variable is not only tedious, but can also lead to misinterpretation of data because of the different interactions that might be overlooked among variables. Statistical optimization allows quick screening for significant variables in a large experimental design while also identifying important roles of each component (Abdel-Fattah et al., 2005; Rathnasabapathy et al., 2009). In our study, we used an integrated statistical approaches incorporating Plackett–Burman and Box–Behnken designs to optimize media components for GSH production by *L. plantarum*.

## Materials and Methods

### Test Microorganisms

Twenty-five *L. plantarum* isolates were previously collected from fermented milk and identified by API-50CHL (Al-Madboly and Abdullah, 2015).

### Screening for the Production of Reduced Glutathione (GSH) among *L. plantarum* Isolates

Test isolates were screened for the intracellular GSH using cell lysates.

#### Preparation of Cell Lysates

Overnight cultures grown on De Man-Rogosa-Sharpe (MRS) agar at 37°C were used to inoculate 50-ml Falcon tubes containing MRS broth and were incubated under anaerobic conditions until log phase. Bacteria were harvested by centrifugation (5,000 ×*g*) at 4°C for 15 min. Next, the pellet was washed twice with phosphate buffered saline (PBS, pH 7.4) and then re-suspended in PBS. The cell suspension was disrupted using an ultrasonicator (Branson Sonic Power, USA) in an ice bath. Cellular debris was removed by centrifugation (10,000 ×*g* for 10 min at 4°C). Following this step, cell lysates were used to quantify total protein content and GSH levels. Each measured parameter represents results obtained from three separate experiments (Zhang et al., 2007).

#### Determination of GSH Content

A solution of cold (4°C) 320 mM sulfosalicylic acid, 28 mM L-ascorbic acid, and 4 mM EDTA was added to cell lysates for protein precipitation. Precipitated proteins were removed by centrifugation (27,000 ×*g*, 15 min, 4°C), and the clear supernatants were used to determine the GSH concentration through a GSH assay kit (Biodiagnostics Cat no. TA2511, Egypt) based on the colorimetric method described by Beutler et al. (1963). This reaction depends on the reduction of 5,5′dithiobis (2-nitrobenzoic acid DTNB), which is dissolved in 25 mM PBS, pH 7.0, by the reduced glutathione to give a yellow product measured at 405 nm. GSH contents were expressed as μM of GSH per gram. Each measurement was repeated in triplicate, the means and standard deviations were calculated.

### Molecular Identification of the GSH Over-Producing Isolate

Identification of the GSH over-producing isolate was further confirmed by 16S rRNA gene sequencing. Briefly, growth of an overnight culture at 37°C of the selected isolate (L_7_) was used for the preparation of genomic DNA that was extracted using GenJET Genomic DNA purification Kit [Thermo Scientific, (EU) Lithuania] according to the manufacturer’s instructions. The target isolate was identified by 16S rRNA gene sequencing using universal primers AGAGTTTGATCMTGGCTCAG and TACGGYACCTTGTTACGACTT. The PCR mixture consisted of 10 pmol of each primer, 10 ng of chromosomal DNA, 20 mM dNTPs, and 2.5 U of Taq polymerase in 50 μl of polymerase buffer (Fermentas, Germany). The PCR was run for 34 cycles at 94°C for 1 min, 55°C for 1 min, and 72°C for 10 min. The 16S rRNA gene fragment (1450 bp length) was sequenced. Multiple sequence alignment and molecular phylogeny were performed using BioEdit 7.0.5.3 and TreeViewX.

### Effect of the Growth Phase on the GSH Content of the Selected Isolate

Glutathione content was determined as previously described in the cell lysate of *L. plantarum* isolate (L_7_) following growth in MRS broth at 37°C under anaerobic conditions. Growth curve was prepared by measuring the optical density (OD) of the cells at 660 nm spectrophotometrically at 0, 2, 4, 6, 8, 10, 24, 36, 48, 60, and 72 h in a disposable cuvette (Zhang et al., 2007; Olson and Aryana, 2012).

### Optimization of GSH Production

#### Plackett–Burman Screening Design and Statistical Analysis of the Data

A Plackett–Burman experimental design (Plackett and Burman, 1946) was used to evaluate the significance of multiple media compositions for production of GSH. Fourteen parameters were tested at two levels, -1 for the low and +1 for the high as shown in **Table 1**, based on a Plackett–Burman matrix design (**Table 2**). This was representing two level factorial design and allowing the investigation of n-1 variables in at least n-experiments. In this work, a design matrix with 16 trials was used to study the selected 14 variables (Ali et al., 2013). The Plackett–Burman experimental design was based on a first-order model: Y = β_0_ + Σ βiXi, where Y is the response (GSH amount), β_0_ is the model intercept, βi is the linear coefficient, and Xi is the level of the independent variable. This model did not describe any interaction among factors, and it was used to determine important factors influencing production of reduced GSH levels.

**Table 1:** Variables and levels used in Plackett–Burman design for screening of culture conditions affecting glutathione (GSH) production.

Variables	-1	+1
- Osmotic stress: sodium chloride (%)	1	5
- Oxidative stress:		
• Bile salt (%)	–	0.5
• Hydrogen peroxide (%)	–	0.05
• Urea (%)	–	0.1
- Detergent: SDS (%)	–	0.05
- Alcohols:		
• Ethanol (%)	–	2
• Butanol (%)	–	1
- Salts: Potassium chloride (%)	–	0.5
- Incubation under aerobic conditions	–	+
- Precursor amino acids (equally mixed combination of glycine, L-cysteine, and glutamic acid, 0.05%)	–	+
- pH	6	8
- Temperature (°C)	30	40
- Incubation time (h)	18	24
- Cooling after incubation at 4°C	–	24 h

**Table 2:** Plackett–Burman experimental design for screening the significant variables affecting the production of glutathione.

Trial no.	Sodium chloride	Bile salt	H_2_O_2_	SDS	Ethanol	KCl	Urea	Butanol	Aerobic incubation	Amino acids	pH	Temperature	Incubation time	Cooling	GSH μM/g
1	1	1	-1	1	-1	-1	-1	1	1	1	-1	-1	1	-1	260
2	-1	1	1	-1	1	1	1	-1	1	1	-1	-1	-1	1	245
3	1	-1	1	1	-1	-1	-1	1	-1	1	1	-1	-1	-1	1147
4	-1	1	-1	1	1	1	1	-1	1	-1	1	1	-1	-1	917
5	-1	-1	1	-1	1	1	-1	1	-1	1	1	1	1	-1	956
6	-1	-1	-1	1	-1	-1	1	-1	1	-1	-1	1	1	1	1147
7	1	-1	-1	-1	1	1	1	1	-1	1	1	-1	1	1	260
8	1	1	-1	-1	-1	-1	-1	1	1	-1	-1	1	-1	1	275
9	1	1	1	-1	-1	-1	1	-1	1	1	1	-1	1	-1	860
10	-1	1	1	1	-1	-1	-1	1	-1	1	-1	1	-1	1	443
11	1	-1	1	1	1	1	-1	-1	1	-1	1	-1	1	-1	199
12	-1	1	-1	1	1	1	-1	-1	-1	1	1	1	-1	1	153
13	1	-1	1	-1	1	1	1	-1	-1	-1	-1	1	1	-1	1338
14	-1	1	-1	1	-1	-1	1	1	-1	-1	1	-1	1	1	122
15	1	-1	1	-1	1	1	1	1	1	-1	-1	1	-1	1	153
16	-1	-1	-1	-1	-1	-1	-1	-1	-1	-1	-1	-1	-1	-1	122

To prepare the optimization media, *L. plantarum* isolate (L_7_) was grown overnight in MRS agar and inoculated into 500 ml of MRS broth before incubation at 30°C for 18 h until exponential growth reached an OD of approximately 1.2 at 650 nm (OD_660_). Cells were harvested by centrifugation at 5,000 ×*g* for 15 min, washed twice with 20 mM sterile phosphate buffer, pH 7, standardized to obtain a final OD_660_ of 2, and then used to inoculate each trial under test. The trials were prepared as follows: 52 g of MRS powder was suspended in 1 L of distilled water and put in a boiling water bath for 10 min; 80 ml was dispensed into 150 ml flasks, and each trial was prepared according to the concentrations mentioned in **Table 1** and the matrix designed in **Table 2**. Flasks were autoclaved except for heat sensitive materials like precursor amino acids that were filter sterilized then added following autoclaving. Each flask was inoculated with the selected isolate then the final volume was completed to 100 ml with sterile MRS broth and incubated either anaerobically in Genbox jars (BioMérieux SA, France) using AnaeroGen bags (Oxoid, England) or aerobically according to **Table 2**. The GSH data were subjected to statistical analysis, where Essential Experimental Design free software was used for the data analysis, determining the coefficients, and the polynomial model reduction (Steppan et al., 1999).

#### Box–Behnken Experimental Design and Statistical Analysis of Data

To describe the nature of the response surface in the experimental region and to identify the optimum conditions for GSH production, a Box–Behnken design was applied (Box and Behnken, 1960). Each significant variable was studied in three levels coded -1, 0, and +1 for low, middle, and high values, respectively, as recorded in **Table 3**. The design matrix consisted of 13 trials was used to study the most significant variables affecting GSH production as shown in **Table 4**. To predict the optimal point, a second order polynomial function was fitted to correlate the relationship between the independent variables and the response (GSH amount), for three factors. The equation was: Y = β_0_ + β_1_X_1_ + β_2_X_2_ + β_3_X_3_ + β_12_X_1_X_2_ + β_13_X_1_X_3_ + β_23_X_2_X_3_ + β_11_X_1_^2^ + β_22_X_2_^2^ + β_33_X_3_^2^, where Y is the predicted response, β_0_ is the model constant, X1, X2, and X3, are the independent variables, β_12_, β_13_, and β_23_ are the cross product coefficients, and β_11_, β_22_, and β_33_ are the quadratic coefficients. Microsoft Excel 2007 was used for the regression analysis of the experimental data. The quality of fit of the polynomial model equation was expressed by a coefficient of *R*^2^ determination. Experiments were performed in triplicate, and the mean values are given. The optimal production value was estimated using the Solver function of the Microsoft Excel tools.

**Table 3:** Levels of the selected variables used in Box–Behnken for optimization of GSH production.

Variables	-1	0	+1
Amino acids (%)	0.0125	0.025	0.05
Temperature (°C)	35	40	45
Urea (%)	0.02	0.1	0.5

**Table 4:** Box–Behnken design of GSH production by *L. plantarum* as influenced by amino acids, temperature, and urea.

Trail no.	Precursor amino acids	Temperature	Urea	Experimental GSH (μM/g)	Predicted GSH
1	0	-1	-1	82.34	108.61
2	0	1	-1	129.01	110.84
3	0	-1	1	156.77	174.94
4	0	1	1	194.51	168.25
5	-1	-1	0	239.01	215.41
6	-1	1	0	305.76	326.59
7	1	-1	0	550.02	529.19
8	1	1	0	389.93	413.53
9	-1	0	-1	426.33	423.67
10	-1	0	1	512	517.43
11	1	0	-1	661.35	655.92
12	1	0	1	683.24	685.90
13	0	0	0	1599.06	1599.06

## Results and Discussion

**FIGURE 1:**
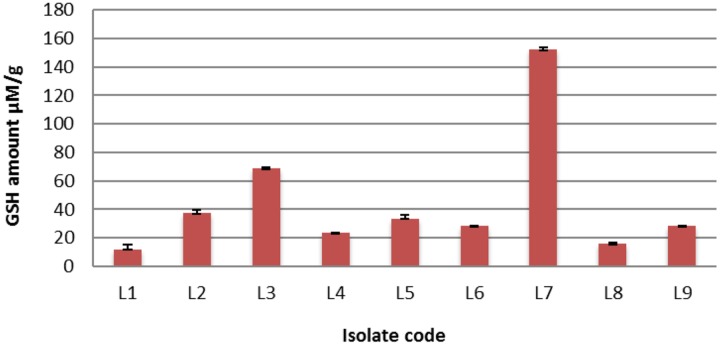
**The detected quantities of reduced glutathione (GSH) among different *Lactobacillus plantarum* isolates**.

Twenty-five *L. plantarum* isolates were previously isolated from fermented milk (Al-Madboly and Abdullah, 2015) and screened for intracellular GSH contents. It was found that only 9 out of 25 (36%) *L. plantarum* isolates were positive for GSH production, meaning that GSH production was strain specific. The range of reduced GSH varied between 12.22 and 152.18 μM/g. Interestingly, isolate L_7_ over-produced GSH (152.18 μM/g protein) as shown in **Figure 1**. It is noteworthy that isolate L_7_ has the highest GSH amount reported for a bacterium (Fernandes and Steele, 1993; Wiederholt and Steele, 1994; Yoon and Byun, 2004; Pophaly et al., 2012).

**FIGURE 2:**
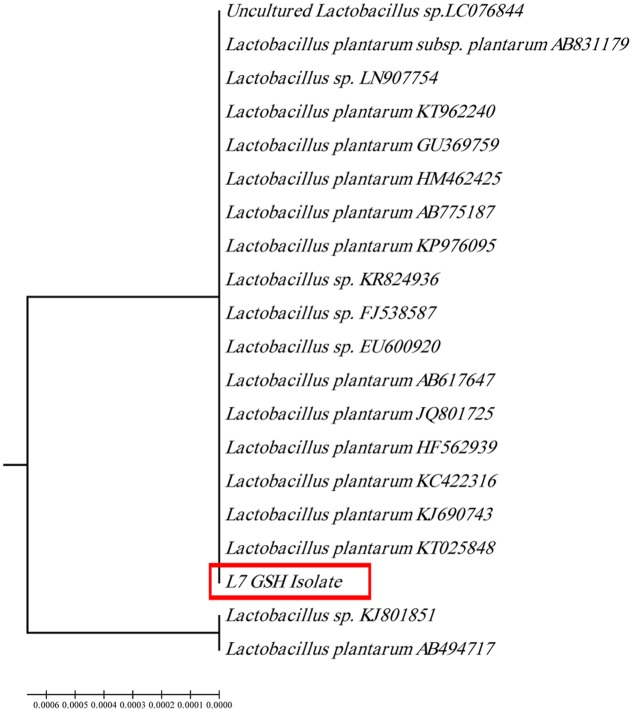
**Phylogenetic tree of the isolate coded (L_7_ GSH) showing the position of this isolate among the selected Lactobacilli based on 16Sr RNA sequences from NCBI**.

**FIGURE 3:**
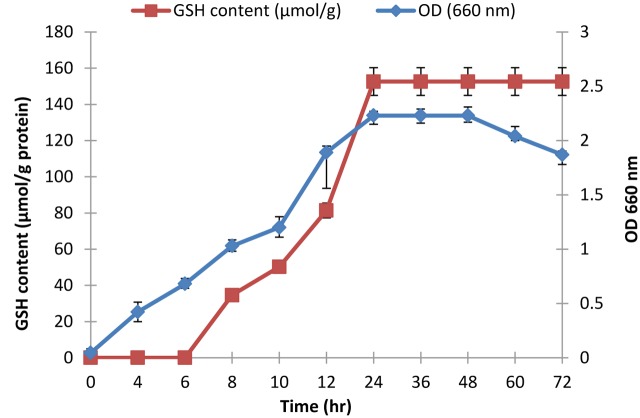
**Time-course of glutathione production by *L. plantarum* along with the biomass**.

The selected isolate (L_7_) was confirmed as *L. plantarum* by 16S rRNA sequencing. The 16S rRNA gene sequence was deposited in the GeneBank under the accession number KU720558. A phylogenetic tree was constructed using the Clustal X 2.0.11 program^1^, which showed that the L_7_ isolate is related to *L. plantarum* with 99% identity as shown in **Figure 2**. The time-course of GSH content along with the biomass measurements of *L. plantarum* isolate (L_7_) were presented in **Figure 3**. The highest biomass (2.23) was detected at 24 h of incubation. Maximal GSH content (152.61 μM/g) was detected at the end of the exponential growth phase and then remained constant till the end of the growth cycle. As a result, the action of some peptidases excreted into the medium during growth was canceled. Similarly, Yamada et al. (1984) reported that GSH was detectable in the control culture media of *Candida tropicalis* during growth. In addition, Yoon and Byun (2004) stated that *L. casei* HY 2782 showed high intracellular GSH levels reached 25.15 μM/g after 24 h of cultivation. As incubation proceeded, GSH levels tended to decrease until reached 5 μM/g after 72 h suggesting synthesis and release of certain peptidases into the medium.

The ultimate target of the biotechnological production of GSH is to achieve a high total GSH concentration through increasing the intracellular GSH content and cell density (Li et al., 2004). This could be achieved through selection of certain nutrient components in the culture media. It was reported that the best nitrogen sources increased the growth rate of *S. cerevisiae* were peptone and yeast extract. Moreover, their presence in the media enhanced the GSH productivity (Liu et al., 1999). Additionally, Shimizu et al. (1991) mentioned that the use of glucose as a sole carbon source at 1.5% was associated with marked increase in the biomass of *S. cerevisiae* as well as the GSH production. Furthermore, 0.15% magnesium sulfate was foremost important salt that could stimulate GSH production and cell growth. Lee et al. (2010) reported similar effect for magnesium sulfate on *L. acidophilus* A12. In the present work, all the above media components were already included in MRS in addition to other ingredients that are sufficient to enhance the cell density and GSH productivity. That is why we used MRS broth as a basal medium in our study. Yoon and Byun (2004) studied the relationship between the type of the media and the cellular GSH levels in *L. casei* HY 2782 and they found significant high GSH levels when MRS was used.

In preliminary experiments, we evaluated the effects of different separate stressful factors on the GSH production by L_7_ isolate as well as its biomass. We found that 0.1% ammonium persulfate, 2% MgCl_2_, 0.5% EDTA, 2% CaCl_2_, and 1% ZnSO_4_ and pH 4 decreased the production of GSH with slight decrease or no effect on the cell density when they were added to the basal medium. On the other hand, 0.5% bile salt, cooling at 4°C following incubation, 5% NaCl, pH 8, 0.05% SDS, 0.025% H_2_O_2_, 1% butanol, 1% urea, 2% ethanol, and 1% precursor amino acids increased GSH production with enhanced survival or no effect on the biomass compared to the control (preliminary data not shown). It was reported that *L. plantarum* from different niches could tolerate osmotic, oxidative, acid, alkaline, detergent and starvation stressors with a probability of stress-induced GSH production (Milesi et al., 2008; Pophaly et al., 2012). Cells of *Lc. lactis* subspecies *cremoris* SK11 presented 30% higher GSH levels when incubated under aerobic conditions (Li et al., 2003). Additionally, Zhang et al. (2009) studied osmoadaptation by *Lc. lactis* and they found that it was able to resist up to 5 M NaCl upon supplementation of GSH. Although cold stress decreased the levels of GSH produced by bacterial cells, supplementation of GSH to the medium could replenish the loss (Zhang et al., 2010). Furthermore, synthesis and accumulation of GSH by lactic acid bacteria as well as yeast could be correlated to their ability to combat reactive oxygen species such as H_2_O_2_ (Ubiyvovk et al., 2011; Pophaly et al., 2012). Accordingly, we decided to use a statistically based experimental design (Plackett–Burman) to screen for significant variables, among the above mentioned, influencing GSH production by the *L. plantarum* (L_7_) isolate. In addition, levels of the significant variables were further optimized using Box–Behnken design.

**FIGURE 4:**
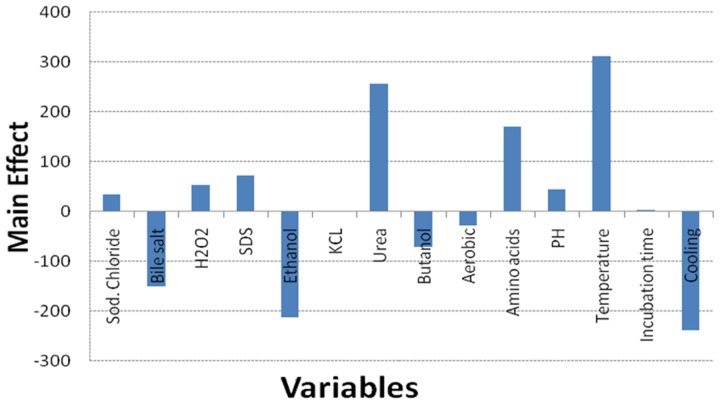
**Effect of culture conditions on the GSH production by *L. plantarum* isolate based on Plackett–Burman design results**.

Regarding the results of the Plackett–Burman design, there was a variation in the amount of GSH produced upon applying different trials of the design matrix as shown in **Table 2**. It ranged between 122 and 1338 μM/g. The main effects of the tested variables on GSH were calculated and illustrated in **Figure 4**. It showed that sodium chloride (5%), hydrogen peroxide (0.05%), sodium dodecyl sulfate (0.05%), urea (0.1%), precursor amino acids (0.05%), temperature (40°C), incubation time (24 h) and pH 8 stimulated the GSH production. Among these, the most significant variables increasing the productivity were 0.05% precursor amino acids, 0.1% urea and incubation at 40°C. Liang et al. (2008) reported that addition of the three precursor amino acids (glutamic acid 37 mM, glycine 35 mM, cysteine 32 mM) to the culture media of *C. utilis* enhanced the glutathione production dramatically. Springael and Penninckx (2003), reported that urea was an important nitrogenous source for *S. cerevisiae* growth as well as a strong oxidizing agent that was able to stimulate GSH production at a concentration of 0.1%. Additionally, the optimal temperatures for cell growth and glutathione production were different. Many researchers have tried to improve the microbial production of glutathione through temperature adjustment (Shimizu et al., 1991; Alfafara et al., 1992; Li et al., 1998; Wei et al., 2003). In the present work, the quality of fitting model equation was determined with an *R*^2^ of 0.8353. The model equations for the GSH yield: Y_GSH_ = 537.3125 + 169.650611210532 (precursor amino acids) + 310.851847691976 (temperature) + 254.802687529275 (urea).

In the present work, Box–Behnken design determined three levels for each significant independent variable that could optimize GSH production as presented in **Table 3**. The experimental results showed GSH levels that were nearly predicted and the highest amount produced was 1599.06 μM/g as recorded in **Table 4**. Masip et al. (2006) reported that *L. lactis* transformed with a plasmid expressing *Escherichia coli gshA* and *gshB* genes resulted in a high intracellular GSH concentration, up to 140 mM, following addition of 5 mM L-cysteine. In our study, the Box–Behnken design showed produced GSH levels of 160 mM, which is the highest concentration ever reported for a bacterial system, particularly because it was obtained by endogenous production and not transformation.

**FIGURE 5:**
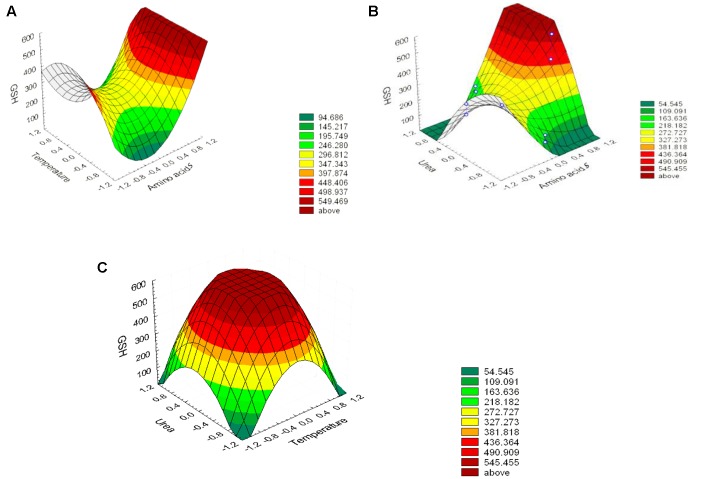
**Three-dimensional response surfaces representing the effect of the three significant parameters on the GSH production by *L. plantarum* L_7_ isolate.** When the effect of two parameters was plotted, the remaining one was set at central level. **(A)** urea and precursor amino acids, **(B)** temperatures and precursor amino acids, **(C)** temperatures and urea. Outside (–1, 1) is a result of extrapolation of the model.

The effect of the amino acid precursors, temperature and urea on GSH production was represented as surface plots as shown in **Figure 5**. These plots were generated to study the effects of the three significant parameters on the GSH yield as a function of two factors while the remaining variable was set at its zero level. It was obvious from the plots that GSH yield was sensitive to alterations in the test variables. Significant increase in the GSH yield was observed due to the combined effect of urea and precursor amino acids (**Figure 5B**), and urea and temperature (**Figure 5C**). However, the interaction between precursor amino acids and temperature (**Figure 5A**) was relatively less significant which was consistent with the conclusion of Wen et al. (2005) and Liang et al. (2008). Glutamic acid is a primary metabolite that could be synthesized by *L. plantarum* and replenish the requirement for GSH production and this might explain why the interaction between precursor amino acids and temperature was comparatively less significant (Zareian et al., 2012). The optimal levels of the three components obtained from the maximum point of the polynomial model were found to be amino acids (0.0281%), urea (0.1916%), and temperature 40°C. Following data analysis, the composition of the optimized medium was MRS broth supplemented with NaCl (5%), H_2_O_2_ (0.05%), SDS (0.05%), precursor amino acids (0.0281%), and urea (0.1916%). The pH of the media was adjusted to 8 and incubated for 24 h at 40°C. Verification of the model was done by conducting the experiment using the optimized medium. It resulted in GSH yield of 1610.22 μM/g which is close to the predicted response (1599.06 μM/g) indicating the validity of the model. GSH production in the optimized medium was 10-fold (851%) higher than what was produced using the basal medium alone. Alfafara et al. (1992) reported that the addition of amino acids was required for GSH production even though sugar was the major substrate consumed in fermentative production of GSH. Furthermore, a stimulatory effect of 9 mM L-cysteine on GSH production was observed in recombinant *E. coli* (Li et al., 1998), where the total GSH concentration and the intracellular GSH content increased by 40 and 100%, respectively. In addition to L-cysteine, several other substrates were found to stimulate GSH production (Liang et al., 2008), ethanol on *S. cerevisiae* (1.4-fold increase; Kyowa Hakko Kogyo Co., Ltd, 1984), amino acids supplements on *S. cerevisiae* (3.4-fold increase; Watanabe et al., 1986), *p*-amino benzoic acid on *Hansenula capsuleita* (94% increase; Kinoshita et al., 1986), and sodium lactate on *S. cerevisiae* (82% increase; Hirakawa et al., 1985). Additionally, Liang et al. (2009) found that lower pH value favored the growth of *C. utilis* but decreased GSH production, whereas higher pH values promoted GSH production and inhibited cell growth. Accordingly, they altered the pH and found that to be effective. In our study, adjusting pH as high as 8 promoted GSH production as shown in **Figure 4**. In addition, the selected strain L_7_ showed resistance to pH 8 and reached a high OD (data not shown). On the other hand, Zhang et al. (2007) and Ubiyvovk et al. (2011) reported that low pH values resulted in higher GSH levels.

## Conclusion

A GSH over-producing food-grade microorganism such as *L. plantarum* (KU720558) was previously isolated and used in the present study. Fermentative production of GSH was successfully optimized through subjecting the strain to a matrix of stressors using Plackett–Burman and Box–Behnken statistical designs. Our study showed 10-fold increase (851%) in the GSH yield. This design offers significant opportunity for GSH production by *L. plantarum* recommending the use of the optimized media and conditions.

## Author Contributions

LA-M conceived the experiments, LA-M, SA, and EK conducted the experiments, SA analyzed the results. All authors reviewed the manuscript.

## Conflict of Interest Statement

The authors declare that the research was conducted in the absence of any commercial or financial relationships that could be construed as a potential conflict of interest.

## Footnotes

1 http://www.softpedia.com/get/Science-AD/Clustal-X.shtml
